# Gastrointestinal Manifestations in Mucopolysaccharidosis Type III: Review of Death Certificates and the Literature

**DOI:** 10.3390/jcm10194445

**Published:** 2021-09-27

**Authors:** Sophie Thomas, Uma Ramaswami, Maureen Cleary, Medeah Yaqub, Eva M. Raebel

**Affiliations:** 1 The Society for Mucopolysaccharide and Related Diseases, MPS House, Amersham HP7 9LP, UK; 2 Lysosomal Disorders Unit, Royal Free London NHS Foundation Trust, London NW3 2QG, UK; uma.ramaswami@nhs.net; 3 Great Ormond Street Hospital for Children NHS Foundation Trust, Great Ormond St., London WC1N 3JH, UK; Maureen.Cleary@gosh.nhs.uk; 4 Rare Disease Research Partners, MPS House, Amersham HP7 9LP, UK; m.yaqub@rd-rp.com

**Keywords:** mucopolysaccharidosis, Sanfilippo syndrome, mortality, gastrointestinal

## Abstract

Background: Mucopolysaccharidosis type III (MPS III, Sanfilippo disease) is a life-limiting recessive lysosomal storage disorder caused by a deficiency in the enzymes involved in degrading glycosaminoglycan heparan sulfate. MPS III is characterized by progressive deterioration of the central nervous system. Respiratory tract infections have been reported as frequent and as the most common cause of death, but gastrointestinal (GI) manifestations have not been acknowledged as a cause of concern. The aim of this study was to determine the incidence of GI problems as a primary cause of death and to review GI symptoms reported in published studies. Methods: Causes of death from 221 UK death certificates (1957–2020) were reviewed and the literature was searched to ascertain reported GI symptoms. Results: GI manifestations were listed in 5.9% (*n* = 13) of death certificates. Median (IQR) age at death was 16.7 (5.3) years. Causes of death included GI failure, GI bleed, haemorrhagic pancreatitis, perforation due to gastrostomies, paralytic ileus and emaciation. Twenty-one GI conditions were reported in 30 studies, mostly related to functional GI disorders, including diarrhoea, dysphagia, constipation, faecal incontinence, abdominal pain/distension and cachexia. Conclusions: GI manifestations may be an under-recognized but important clinical feature of MPS III. Early recognition of GI symptoms and timely interventions is an important part of the management of MPS III patients.

## 1. Introduction

Mucopolysaccharidosis type III (MPS III, Sanfilippo disease) is a rare autosomal recessive lysosomal storage disorder (LSD) caused by the accumulation of glycosaminoglycan (GAG) heparan sulfate due to the deficiency of specific enzymes responsible for its degradation [1]. Four distinct MPS III subtypes have been recognized, depending on the enzyme deficiency: MPS III type A (heparan N-sulphatase, sulfamidase; MIM #252900), type B (α-N-acetylglucosaminidase; MIM #252920), type C (acetyl-CoA α-glucosaminide N-acetyltransferase; MIM #252930), and type D (N-acetylglucosamine 6-sulfatase; MIM #252940) [1,2]. Overall prevalence is estimated at 1–9 births per one-million population, with subtypes varying geographically and incidence of subtypes A and B being the most diagnosed in Europe [1,3]. UK prevalence has been estimated at approximately 1.21 per 100,000 live births [4].

MPSIII is a life-limiting condition prevalently characterized by a progressive severe deterioration of the central nervous system (CNS), including neurocognitive and behavioural decline [5]. Following an initial normal birth and development, the disease tends to progress through three main phases: the first phase usually begins at 1–3 years of age and is characterized by delayed cognitive development, specially speech delay; the second phase starts between 3–4 years of age and it is marked by the beginning of cognitive decline with challenging behaviour, including aggression, hyperactivity and sleep disturbance; the third stage, usually from 10 years of age onwards, is quieter as behavioural difficulties disappear but there is a rapid loss of cognitive processes and motor functions, including walking, swallowing and the development of seizures and pyramidal symptoms [1,2,6,7,8]. Patients in this last phase tend to be immobile, fed by enteral tube and incontinent, becoming fully dependent on care. MPS III also presents with somatic symptoms, but those are relatively less severe if compared to other MPS disorders [5,9,10]. Associated somatic signs and symptoms can include mild facial dysmorphology, hirsutism, recurrent ear, nose and throat (ENT) infections, frequent upper respiratory infections, umbilical and inguinal hernias, coarse hair, hepatomegaly, splenomegaly, recurrent diarrhoea, constipation, hearing loss, scoliosis, odontoid hypoplasia, femoral head osteonecrosis, cardiac disease and abnormal dentition [5,6,7,9,11,12,13,14,15,16].

Life expectancy for individuals with MPS III varies greatly but death usually occurs between the second and third decades of life, even though survival into later decades has been reported, depending on the disorder’s subtype and the phenotype (severe and attenuated forms) [11,17,18]. Pneumonia and respiratory tract infections have been reported as the most common causes of death (>50%) in MPS III individuals with types A, B [11,17,18,19] and C [20].

The large differences in disease onset, clinical manifestations and life span between individuals are the result of genetic heterogeneity producing inter- and intra- type variability [21]. Due to the rarity of MPS III and the non-specificity of early-symptoms, early diagnosis of MPS III remains a challenge and median diagnostic delay between initial symptoms and diagnosis can range from 2 years in MPS IIIA [6] to 28 years in MPS IIIB individuals with the attenuated phenotype [18]. Currently, there is no approved disease-modifying therapy for MPS III, with treatment being limited to the management of clinical symptoms and palliative care [2].

The UK Society for Mucopolysaccharide and Related Diseases (MPS Society) is a patient support group providing advocacy and support to individuals and families diagnosed with a MPS or related conditions. Through its work with clinicians, patients, and their families, the MPS Society was made aware of gastro-intestinal (GI) problems presenting in patients with MPS III. Personal communication [ST] with patients and LSD medical specialists from UK metabolic centres revealed GI symptoms and signs are frequently reported, including constipation, diarrhoea, intolerance to feeds, recto-vaginal fistulas, malabsorption of feeds, bowel volvulus, bowel stasis, ulcerative colitis, GI bleeding and pseudo-obstruction, with some individuals having had a diagnosis of irritable bowel syndrome or Crohn’s disease.

To date, GI symptoms have not been acknowledged in previous studies as a primary cause of concern in the MPS III population, however, parents have identified digestive, toileting and feeding issues as an unmet treatment need in MPS III, causing significant concerns and challenges on the child’s family [22]. Determining their incidence and severity is paramount to understand MPS III natural history and disease progression, and to improve the future clinical management of these patients. The aim of this study is to review death certificates of individuals with MPS III to determine the incidence of GI manifestations as a leading or contributing cause of death, and to review GI symptoms and signs reported in published studies of live or deceased individuals with MPS III.

## 2. Materials and Methods

### 2.1. GI Manifestations as a Cause of Death in UK Patients with MPS III

The MPS Society UK holds details of 416 individuals with MPS III in their database. According to the UK Data Protection Act 2018, the General Data Protection Regulation (GDPR) only applies in the UK to living individuals (https://www.legislation.gov.uk/ukpga/2018/12/section/3/enacted (accessed on 20 July 2021)). Data on deceased patients with MPS III were retrospectively extracted, including: name, date of birth, date of death, gender, and type of MPS III, where available. To ascertain the cause of death, death certificates, were obtained.

In the UK, a death certificate includes an exact copy of the cause of death given by a medic on the Medical Certificate of Cause of Death (MCCD) [23]. The cause of death section of UK death certificates is divided into two parts. Part I starts on line (a) with the immediate, direct cause of death, and it is followed on subsequent lines (b and/or c) by the sequence of events or conditions that led to death, until reaching the underlaying cause of death which is the condition that started the fatal sequence. Part II includes other significant conditions that contributed to death but were not related to the disease or condition causing it [23]. Under UK legislation, death certificates are considered public records and duplicate certificates can be requested by anyone if the full name of the deceased individual and date of death is available, to obtain the General Register Office (GRO) index reference number (Births and Deaths Registration Act 1953; (https://www.legislation.gov.uk/ukpga/Eliz2/1-2/20/section/30 (accessed on 20 July 2021)). If the date of death is not available, this can be found in the register if the birth year is available. For individuals ≤16 years of age, the full name of both parents is required, together with the latest registered address. This was available for some members on the database. The MPS Society held copies of death certificates registered from January 1957–March 2006, including some from a previous mortality study [17]. Death certificates from April 2006–October 2020 were obtained, with the last request made on 21 May 2021. Death certificates were reviewed for listed GI manifestations recorded as cause of death. Causes of death for one individual could include different conditions leading to death (e.g., respiratory and gastrointestinal conditions). Data was anonymised and all patients were deceased, hence ethical approval was not required.

### 2.2. GI Manifestations in the Literature

A literature search was conducted in PubMed to ascertain GI symptoms and signs in MPS III patients reported in published studies. The pre-determined search terms ‘Sanfilippo syndrome’ and ‘Mucopolysaccharidosis type III’ were combined using the Boolean ‘OR’ operator. Studies were retrieved if the title/abstract/keyword contained at least one of the terms. The Boolean term ‘NOT’ was used to exclude animal studies. Searches were completed on 20 April 2021. The Rayyan Web app [24] was used to screen titles, abstracts and keywords by two researchers (ER, MY) to identify potential studies for inclusion. Further animal studies were excluded. Full texts of relevant papers were subjected to further scrutiny with final papers being selected based on the inclusion criteria in Table 1. GI symptoms and sigs mentioned within the studies were recorded (ER, MY). GI manifestations affecting the liver, or the mouth, were excluded (e.g., hepatomegaly, abnormal dentition). This literature review only aimed to identify the number of studies reporting GI symptoms.

## 3. Results

### 3.1. GI Manifestations as Cause of Death in Patients with MPS III: Death Certificates

#### 3.1.1. Death Certificates

Records of 240 deceased members with MPS III were found on the MPS Society database. Three records did not include the full names of the individuals and seven were of children ≤16 years old for whom the name of both parents was not available. For three individuals, the date of death was not recorded and alternative searches within the government website did not yield any results. Death records for six individuals were not available from the General Register Office even though full name and date of death were provided. A total of 221 death certificates from deceased individuals were included in this study: 113 death certificates were available from Lavery et al. [17], 24 from the MPS Society UK, and 84 new certificates with full records were obtained.

#### 3.1.2. Demographics of Deceased Patients

Median (IQR) age at death of the 221 individuals was 16.0 (6.4) years (mean 17.7 (±7.4), range 3.2–47.8) and 110 (49.8%) of deceased individuals were female. Type of MPS III and decade at death are shown in Table 2. Dates of birth ranged from April 1946 to August 2004, with dates of death between January 1957 to September 2020. Age at death increased over time from a mean age of 16.5 years (±4.3, *n* = 51) in 1990–1999 to 21.8 years (±9.9, *n* = 50) in 2010–2020. A total of 72.4% (*n* = 160) of deaths occurred during the patient’s second decade of life (i.e., between 10–19 years of age) (Table 2).

#### 3.1.3. GI Manifestations Leading or Contributing to Death on Death Certificates

A total of 5.9% (*n* = 13) of deceased individuals had GI conditions listed on their death certificates: 12 (5.4%) certificates had GI conditions recorded as leading to death (Table 3, Part I) and one as significantly contributing to death (Table 3, Part II). Three deaths were associated with complications of percutaneous endoscopic gastrostomies (PEG) (Table 3). Median (IQR) age at death of these individuals was 16.7 (5.3) years (mean 18.1 (±7.3), range 11.2–39.7) and 38.5% (*n* = 5) were female. Type of MPS III and decade at death are shown in Table 2. Dates of birth ranged from January 1966 to March 2002, with dates of death between January 1981 to August 2018. Nine deaths (76.9%) occurred during the patient’s second decade of life (i.e., between 10–19 years of age). Two patients died in their third decade of life and one in their fourth (Table 2).

### 3.2. GI Manifestations Reported in the Literature

A total of 837 papers were identified in the PubMed search and were subjected to abstract review; of these, 731 were excluded, based on the pre-determined inclusion/exclusion criteria (Table 1). Full-text review was performed on 53 papers, from which 30 reported GI manifestations, both as individual case studies, a case series, or as aggregated data within retrospective/prospective studies.

Twenty-one GI signs and symptoms were reported in the literature, mostly related to functional GI disorders (Table 4). Diarrhoea and dysphagia were commonest (*n* = 16 and 12 studies, respectively), followed by constipation (*n* = 5) and loss of bowel control/ faecal incontinence (*n* = 4). Nine studies reported patients needing a gastrostomy and four studies mention nasogastric/feeding tube.

## 4. Discussion

This is the first retrospective study of individuals with MPS III presenting with significant GI manifestations leading or contributing to death. Our results showed that gastrointestinal complications led or contributed to 5.9% of deaths in this population. Lavery et al. [17] showed that some conditions can be relatively high within the non-pulmonary related deaths. Although pulmonary conditions in our study were listed in 50.7% of death certificates, cardiac arrest led or contributed to 2.3% of deaths, aspiration pneumonia to 3.6%, congestive/cardiac failure to 4.5%, respiratory failure to 5% and epilepsy/seizures to 6.8%, while other conditions had a prevalence of <1% (e.g., renal failure, sepsis). Our results could not be directly compared to those in Lavery et al.’s publication as it was not possible to ascertain how the causes of death had been classified in the study.

Most degenerative disorders can present with feeding problems because of functional decline. By the time MPS III children reach their second decade of life, dysphagia and an increased need for aspiration usually result in the requirement of a nasogastric tube or gastrostomy feeding to avoid chocking and severe debility [19,26]. Indeed, extreme emaciation contributed to the cause of death in one of the individuals in our study and three deaths were a consequence of PEG-related complications. A Dutch study on adult phenotype and natural history of patients with MPS IIIB reported six deaths, including a 51 old male who died from complications after gastrostomy replacement and a 68 year old female who died of cachexia a year after developing difficulties with swallowing [33]. Review of the death certificates indicated that, besides deaths related to feeding problems, other GI conditions play a role in MPS III mortality, including GI failure, GI bleed, gastroenteritis and paralytic ileus. Information on causes of death in MPS III patients is limited in the literature with numerous studies stating the number of deceased individuals but not their cause of death, suggesting that GI manifestations as a cause of mortality may be underreported.

Death certificates showed that the trend for increased survival reported by Lavery et al. [17] continued in 2010 to 2020. Improved medical care (e.g., enteral feeding such as gastrostomies), supportive and multidisciplinary care, better awareness of the disease in the community leading to earlier diagnosis and detection of attenuated cases, and referral of patients to specialist centres, are possible reasons for this increase in life expectancy [17,37].

Inflammation may be a contributor to neurodegeneration in LSDs and problems related to inflammatory bowel disease (IBD) in some LSDs have been reported. In Fabry Disease, IBD-like symptoms can include unspecified functional bowel disorder, functional abdominal bloating/distension, irritable bowel syndrome (IBS), diarrhoea, constipation, abdominal pain and early satiety [46,47]. GI signs and symptoms are common in Fabry Disease, possibly due to the accumulation of Gb3 (globotriaosylceramide) substrate in neuronal and muscle intestinal cells, although the physiological processes are not fully understood [47]. GI manifestations in Fabry Disease do not result in mortality. In Niemann Pick C, severe GI symptoms resembling carbohydrate malabsorption leading to extreme weight loss [48] and perianal fistulas indicative of Chron’s disease [49,50] have also been reported. In MPS III, it is speculated that GAGs may infiltrate into the human GI tract, as evaluation of the GI tract in MPS IIIA mice has demonstrated lysosomal GAG accumulation in the lamina propria of the villi of the duodenum, jejunum and ileum, and an increased lysosomal storage in the submucosa throughout the GI system [51]. Autopsy examination of a deceased MPS III patient reported in the literature revealed GAG accumulation and vacuolization in the pyloric ring and the extrinsic nerves of the Auerbach nerve plexuses [27]. Although much focus has been placed on MPS III pathology in the CNS, mouse models have demonstrated lesions in the peripheral nervous system (PNS), with lysosomal storage damage in the myenteric plexus and submucosal plexus, which involve enteric neurons in the GI tract [52]. This effect on the PNS may explain the autonomic abnormality of GI peristalsis in MPS III patients and some of the GI manifestations reported in this study.

Despite GI symptoms not being a well-recognised clinical finding in MPS disorders, non-specific GI symptoms were reported for MPS III individuals in the literature, including abdominal pain and distension, recurrent diarrhoea, reflux, weight loss and vomiting. A mini-review of MPS III [1] described diarrhoea as episodic, with other studies showing recurrent diarrhoea can affect 50–92% of patients with MPS IIIA, B & C [11,18,19,21,34], interspersed with bouts of constipation, frequently present as patients get older [11,18]. Diarrhoea has been well recognized in MPS II children [53]. Loss of bowel control and faecal incontinence was reported in four studies from our review. Although adaptive behaviours in MPS III individuals persist for longer than cognitive functions, loss of bowel control is one of the adaptive behaviours most affected by disease progression [6]. Since these symptoms are non-specific and functional, thus not explainable by structural or biochemical abnormalities, the exact cause of these GI symptoms is still unclear.

It is not currently known if GI manifestations are disease related, or if there are other contributing factors, such as comorbidities or the consequence of side-effects and interactions between the numerous medications prescribed to these patients. For example, intestinal dysmotility caused by neuronal dysfunction and immobility as disease progresses may contribute to the development of fistulas. However, enemas administered to treat constipation can also contribute to the development of fistulas and rectal perforations [54] and the regular use of laxatives to treat constipation has been associated with increased constipation and faecal impaction [55]. Similarly, imodium-based antidiarrheals (e.g., loperamide hydrochloride) are widely and chronically used in these patients to treat diarrhoea and fistulas [56], but GI-related side-effects include constipation, nausea, vomiting, abdominal pain and, in rare circumstances, paralytic ileus [57]. MPS III patients have also been found to be particularly susceptible to extrapyramidal side effects (e.g., dystonia, ataxia) of certain behaviour management treatments (i.e., risperidone, olanzapine, or lamotrigine) [58]. There may be a burden of medication in MPS III patients due to the need to ameliorate symptoms of multiple conditions and polypharmacy, defined as taking more than five medications at any one time, has been identified as a risk factor for developing GI conditions and GI motility delays [59]. A study assessing sleep disturbance in MPS III children lists two participants as taking 5–10 concurrent medications to manage sleep, epilepsy, seizures, pain, GI symptoms and other conditions [60]. The implications of polypharmacy on GI manifestations in MPS III patients have not been studied and warrant further investigation.

A limitation of this study is the use of death certificates, which in 22.6% (*n* = 50) of cases only reported ‘MPS III’, ‘Sanfilippo syndrome’ or ‘MPS’ as the cause of death. In addition, GI conditions contributing to death may not be reflected in death certificates. For example, one death certificate listed aspiration pneumonitis and seven aspiration pneumonia as the cause of death, which may have been initiated by gastroesophageal reflux or by the inhalation of food, drink, vomit or saliva as a consequence of dysphagia. Limitations and advantages on the use of death certificates to determine cause of death in MPS III individuals have been reviewed in Lavery et al. [17]. A further limitation with the use of death certificates and reported cases in the literature is the lack of clinical data and information on the disease journey of these patients, which may have helped with elucidating at what age GI symptoms first presented, how long these symptoms were managed with medication, how long PEGs were required for, and whether symptoms were the consequence of disease progression, the result of long-term medication and surgeries, or an outcome of patients now living longer. Furthermore, only a compilation of GI manifestations mentioned in these studies is included here, further analysis, including the number of cases within case series and GI symptoms reported as aggregated data will be presented elsewhere.

To be able to support MPS III patients and their families with achieving the best quality of life throughout their disease journey, and to be able to characterise these results further, a prospective study using clinical data is warranted to ascertain the prevalence, morbidity and mortality associated with GI manifestations in individuals with MPS III.

## 5. Conclusions

This retrospective study has identified significant GI pathology leading or contributing to the cause of death in individuals with MPS III. GI manifestations may be an under-recognized, but important clinical feature of LSDs, including MPS III. Early recognition of GI symptoms and signs, and timely interventions, are an important part of the management of MPS III patients.

## Figures and Tables

**Table 1:** Inclusion and exclusion criteria of studies.

Inclusion Criteria	Exclusion Criteria
Article in English Patients with a diagnosis of MPS III Case studies, retrospective, or prospective studies Mention of a GI symptom/sign	Full-text paper not available In-vitro, embryonic, pre-natal and molecular level studies

**Table 2:** Characteristics of deceased individuals with MPSIII on death certificates (*n* = 221) and of individuals with GI conditions listed on their death certificate (*n* = 13).

	Deceased Individuals			GI Condition on Death Certificate		
**N**	221			13		
**Gender**						
Males	111			8		
Females	110			5		
**MPS III subtype**	***n* (%)**			***n* (%)**		
A	148	(67.0)		10	(76.9)	
B	34	(15.4)		2	(15.4)	
C	9	(4.1)		—		
Unknown	30	(13.6)		1	(7.7)	
**Age at death (years)**						
Mean age (±SD)	17.7 (±7.4)			18.1 (±7.3)		
Median (IQR)	16.0 (6.4)		16.7 (5.3)			
Range	3.2–47.8		11.2–39.7			
**Range (years)**	***n* (%)**			***n* (%)**		
0–9	9	(4.1)		—		
10–19	160	(72.4)		10	(76.9)	
20–29	36	(16.3)		2	(15.4)	
30–39	12	(5.4)		1	(7.7)	
40–49	4	(1.8)		—		
**Decade at death**	***n* (%)**		**Mean age (±SD)**	***n* (%)**		**Mean age (±SD)**
1950–1959	1	(0.5)	10.7 (—)	—		—
1960–1969	—		—	—		—
1970–1979	4	(1.8)	10.7 (±3.1)	—		—
1980–1989	36	(16.3)	13.4 (±3.6)	2	(16.7)	12.2 (±1.5)
1990–1999	51	(23.1)	16.5 (±4.3)	3	(25.0)	19.1 (±2.7)
2000–2009	78	(35.3)	18.2 (±7.1)	4	(33.3)	21.0 (±12.7)
2010–2020	51	(23.1)	21.8 (±9.9)	4	(33.3)	17.4 (±3.7)

**Table 3:** GI-related conditions (*in italics)* leading or contributing to death on death certificates (*n* = 13). Causes of death recorded on death certificates follow the Medical Certificate of Cause of Death (MCCD) classification [23].

Cause of Death Listed on Death Certificates				
	**Part I:** Disease or Condition Leading to Death			**Part II:** Other Significant Conditions Contributing to Death but not Related to the Disease or Condition Causing It
**Patient**	I(a) Disease or Condition Leading Directly to Death	I (b) Other Disease or Condition, if Any, Leading to I(a)	I (c) Other Disease or Condition, if Any, Leading to I(b)	
1	*Acute haemorrhagic pancreatitis*	—	—	—
2	Respiratory and *gastrointestinal failure*	MPS IIIB	—	Von Willebrand’s Disease
3	*Gut failure*	MPS III	—	ESBL colonisation of chest
4	*Perforation of the bowel*	*Migrated PEG*	MPS III	—
5	*Gastroenteritis*	MPS III	—	Coma
6	*Gastrointestinal bleed*	MPS III	—	—
7	MPS IIIA treated by *palliative gastrostomy with complications*	—	—	—
8	*Aspiration of gastric contents*	MPS III	—	—
9	*Vomiting and aspiration*	MPS III	—	—
10	Sepsis	*Perforated PEG*	—	—
11	*Peritonitis*	*Abdominal abscess*	—	MPS III
12	*Dehydration*	*Paralytic ileus*	MPS III	—
13	Bronchopneumonia	MPS IIIA	—	*Extreme emaciation*

ESBL: extended spectrum beta-lactamase; PEG: percutaneous endoscopic gastrostomy.

**Table 4:** GI manifestations in MPS III reported in the literature.

GI Manifestations	No. of Studies	Studies
**Upper GI tract**		
Gastroesophageal reflux	1	[25]
Gastroenteritis	1	[26]
Pyloric stenosis	1	[27]
Swallowing difficulties (Dysphagia)	12	[19,21,27,28,29,30,31,32,33,34,35,36]
Nasopharyngeal/feeding tube—management	4	[11,18,21,27]
PEG *—management	9	[19,29,32,33,34,36,37,38,39]
Vomiting	1	[34]
Abdominal distention/ protuberant abdomen	3	[28,29,40]
Abdominal pain	2	[30,41]
**Lower GI tract**		
Constipation	5	[10,11,18,33,34]
Diarrhoea	16	[7,10,11,18,19,20,21,26,28,33,34,35,37,41,42,43]
Faecal impaction	1	[31]
Intestinal fistula due to stenosis of pyloric ring	1	[27]
Intestinal lymphangiectasia	1	[41]
Loss of bowel control/faecal incontinence	4	[6,29,41,44]
**General/Others**		
Cachexia	1	[21,33]
Emaciation	1	[28]
Erratic appetite	1	[45]
Excessive weight	1	[10]
Weight loss	1	[31]
Food allergy—multiple	1	[26]

* PEG: percutaneous endoscopic gastrostomy.

## Data Availability

Death certificates are publicly available at the following location: https://www.gov.uk/order-copy-birth-death-marriage-certificate (accessed on 20 July 2021).
